# Identification of pathogenic *Leptospira *species by conventional or real-time PCR and sequencing of the DNA gyrase subunit B encoding gene

**DOI:** 10.1186/1471-2180-6-95

**Published:** 2006-10-27

**Authors:** Andrew T Slack, Meegan L Symonds, Michael F Dohnt, Lee D Smythe

**Affiliations:** 1 WHO/FAO/OIE Collaborating Centre for Reference & Research on Leptospirosis, Centre for Public Health Sciences, Queensland Health Scientific Services, Brisbane, Australia

## Abstract

**Background:**

*Leptospira *is the causative genus of the disease, leptospirosis. Species identification of pathogenic *Leptospira *in the past was generally performed by either DNA-DNA hybridisation or 16s rRNA gene sequencing. Both methods have inherent disadvantages such as the need for radio-labelled isotopes or significant homology between species. A conventional and real-time PCR amplification and sequencing method was developed for an alternate gene target: DNA gyrase subunit B (*gyrB*). Phylogenetic comparisons were undertaken between pathogenic *Leptospira *16srRNA and *gyrB *genes using clustering and minimum evolution analysis. In addition 50 unidentified *Leptospira *isolates were characterised by *gyrB *sequencing and compared with conventional 16s rRNA sequencing.

**Results:**

A conventional and real-time PCR methodology was developed and optimised for the amplification of the *gyrB *from pathogenic *Leptospira *species. Non pathogenic and opportunistic *Leptospira *species such as *L. fainei *and *L. broomi *were not amplified. The *gyrB *gene shows greater nucleotide divergence (3.5% to 16.1%) than the 16s rRNA gene (0.1% to 1.4%). Minimum evolution analysis reveals that the *gyrB *has a different evolution topology for *L. kirschneri *and *L. interrogans*. When the two genes were compared for the identification of the 50 unknown isolates there was 100% agreement in the results.

**Conclusion:**

This research has successfully developed a methodology for the identification of pathogenic *Leptospira *using an alternate gene to 16s rRNA. The *gyrB *encoding gene shows higher nucleotide/evolutionary divergence allowing for superior identification and also the potential for the development of DNA probe based identification.

## Background

Leptospirosis is the zoonotic disease caused by members of the genus, *Leptospira*. They are motile helical spirochaetes that metabolise long chain fatty acids as their carbon source. There are 17 species of *Leptospira *as determined by DNA-DNA hybridisation [1-4]. These species can be further divided into pathogenic, non-pathogenic and opportunistic/possibly pathogenic *Leptospira *with pathogenic species. The pathogenic *Leptospira *include; *L. interrogans*, *L. kirschneri*, *L. santarosai*, *L. weilii*, *L. alexanderi*, *L. borgpetersenii*, *L. *genomospecies 1 and *L. noguchii*. The non pathogenic *Leptospira *include: *L. biflexa*, *L. meyeri*, *L. wolbachii*, *L. *genomospecies 3, *L. *genomospecies 4, *L. *genomospecies 5 and opportunistic/intermediate pathogens *Leptospira *include *L. broomi*, *L. fainei *and *L. inadai *[3]. The grouping of the last three species as opportunistic or possible pathogens is due to the lack of information on the pathogenicity of the species, different phenotypic characteristics compared to the pathogenic *Leptospira *and also the limited number of reports of these species involvement in human leptospirosis.

Before molecular techniques such as DNA-DNA hybridisation or 16s rRNA gene sequencing became available, speciation of the genus *Leptospira *was limited to the classifications of pathogenic (*L. interrogans *sensu lato) or saprophytic (*L. biflexa *sensu lato) and was performed using phenotypic tests such as growth at 13°C/30°C or growth in the presence of a chemical such as 8-Azaguanine [5]. These tests can take up to 28 days to complete and the results can vary within a species [2]. Since the introduction of molecular techniques, the identification of *Leptospira *species has generally been performed using either DNA-DNA hybridisation [2,4] or 16s rRNA gene sequencing. Both of these methods have inherent disadvantages; DNA-DNA hybridisation is laborious and requires the use of radio-labelled isotopes [6] and the 16s rRNA gene has significant sequence homology between species which requires the majority of the gene to be sequenced for a definitive *Leptospira *identification. As an alternative target to 16s rRNA for species identification, the DNA Gyrase Subunit B gene (*gyrB*) has been successfully used for species identification in a wide variety of bacterial genera [7-13]. More recently the *gyrB *gene has been used for the identification of *Leptospira borgpetersenii *isolates from the Amami Islands [14] though this study used universal *gyrB *primers and only conducted limited phylogenetic analysis.

This paper reports the development of a molecular technique for the identification of pathogenic *Leptospira *species using conventional or real-time PCR amplification and sequencing of a partial fragment of the *gyrB *gene. The method was then used to ascertain *gyrB *sequences from representative reference strains of the eight pathogenic species. These sequences were used for phylogenetic and evolutionary comparisons between the species themselves and also between the *gyrB *gene and 16s rRNA gene. To highlight the potential value of the *gyrB *as an alternate identification gene, a blind trial was conducted between the two gene targets to identify previously uncharacterised clinical *Leptospira *isolates.

## Results and Discussion

### *gyrB *Amplification

**Figure 1:**
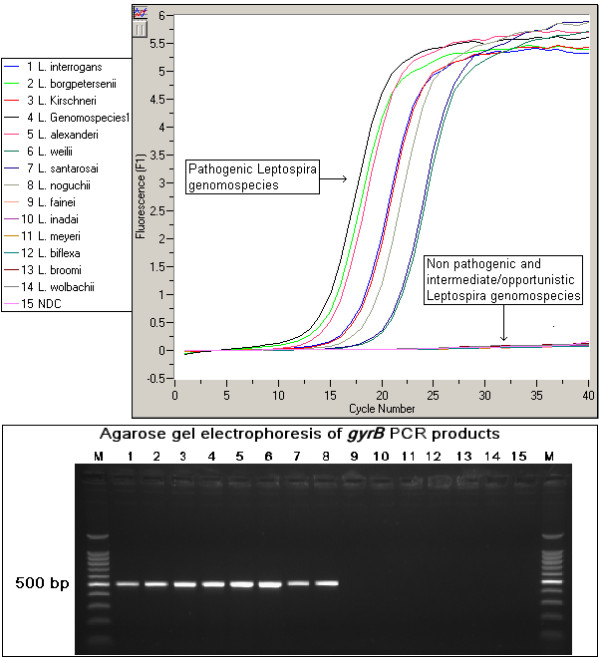
Real-time PCR amplification of the *gyrB *gene from 14 *Leptospira *genomospecies. Agarose gel electrophoresis of the PCR products from the PCR are also shown below. Lanes: M, 100 bp DNA ladder (Promega); 1, *L. interrogans *sv. Australis; 2, *L. borgpetersenii *sv. Ballum; 3, *L. kirschneri *sv. Cynopteri; 4, *L. *genomospecies 1 sv. Pingchang; 5, *L. alexanderi *sv. Manzhuang; 6, *L. weilii *sv. Cellodoni; 7, *L. santarosai *sv. Shermani; 8, *L. noguchii *sv. Cristobali; 9, *L. fainei *sv. Hurstbridge; 10, *L. inadai *sv. Aguarana; 11, *L. meyeri *sv. Semeranga; 12, *L. biflexa *sv. Patoc; 13, *L. broomi *5099T; 14, *L. wolbachii *sv. Codice; 15, No DNA control.

**Figure 2:**
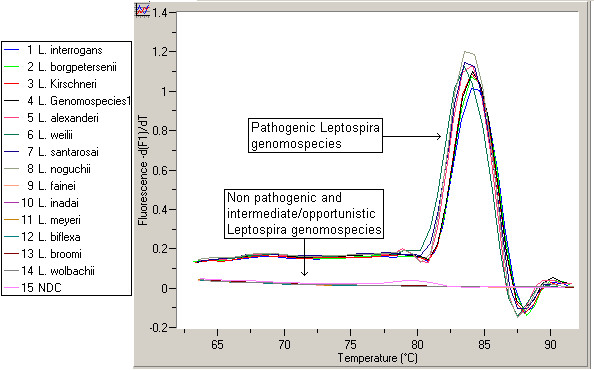
Melting curve analysis of the *gyrB *gene using Sybr green detection.

Using conventional and real-time PCR we were able to amplify a 504 bp product from the eight pathogenic *Leptospira *species: *L. interrogans*, *L. borgpetersenii*, *L. weilii*, *L. santarosai*, *L. alexanderi*, *L. *genomospecies 1, *L. noguchii *and *L. kirschneri*. No PCR products were amplified from the representatives of non-pathogenic species *L. biflexa*, *L. meyeri *or from the pathogenic/intermediate species such as *L. inadai*, *L. fainei *or *L. broomi *(Figure 1 and 2). The development of both conventional and real-time PCR methodologies for the amplification of the *gyrB *gene enables this method to be instituted at the majority of laboratories and is not dependant on having relatively expensive real-time PCR equipment. The advantage of using real-time PCR over conventional PCR is that it is quicker (amplification is completed in less than an hour) and there is no need to perform agarose gel electrophoresis or capture the gel image. Confirmation of the *gyrB *gene amplification was performed using the melting curve analysis on the LightCycler instrument. The Tm of the *gyrB *PCR product was found to be between 83.4°C and 84.8°C (Figure 2). Cycle sequencing of the *gyrB *PCR product and comparison of the DNA sequences enabled species specific identification. The *gyrB *DNA sequences of the reference strains were deposited on GenBank (Table 1).

The amplification of only the pathogenic species from the genus *Leptospira *has created an assay which has a wide potential in this field of research. For example, the *gyrB *PCR could be use to identify pathogenic *Leptospira *from cultures or identify *Leptospira *isolates that have been overgrown with bacteria or fungi. Additionally it would be possible to apply this method to clinical samples that contain high concentrations of *Leptospira *organisms such as kidney tissue. The non-culture identification of pathogenic *Leptospira *would be difficult without the use of specific *gyrB *primers as universal *gyrB *primers such as those used by Kawabata *et al*. [14] would amplify DNA from all bacteria present in a sample.

The potential of this assay must be balanced by three apparent limitations. Firstly the lack of sensitivity of conventional detection methodologies would not enable this test to be used in diagnosis of human infections where there are generally only low levels of *Leptospira *in the blood. Secondly, potentially pathogenic species such as *L. fainei*, *L. inadai *or *L. broomi *are not amplified, and therefore could be missed or excluded during molecular investigations. Finally, if a culture or sample contained two different *Leptospira *species then it would result in a mixed sequencing result, requiring use of DNA cloning and multiple sequencing reactions. The ultimate evolution of this method would be through the use of specific DNA probe detection either through Taqman/FRET probe in a real-time PCR guise or through a more conventional chemical-luminescent detection format.

### Comparative phylogenetic analysis

**Figure 3:**
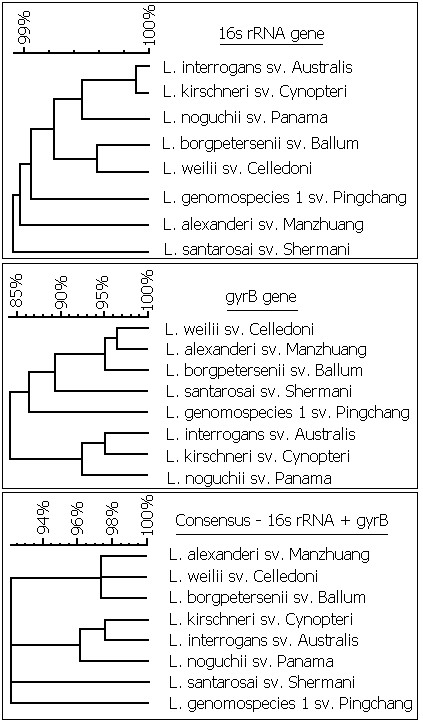
Global cluster analysis of the 16s rRNA gene, *gyrB *and consensus DNA sequences performed using the UPGMA algorithm.

Multiple alignments of the DNA sequences allowed phylogenetic comparisons between the species and between the *gyrB *gene and 16s rRNA gene to be performed. Global clustering (Figure 3) and similarity matrix analysis (Table 2 and 3) was performed using the aligned sequence data and the Unweighted Pair Group Method with Arithmetic Mean (UPGMA) algorithm for the 16s rRNA, *gyrB *and a consensus of both genes to examine the level of relatedness between the pathogenic species. 16s rRNA and *gyrB *genes show significant difference in total relatedness as shown by the percentage scale in Figure 3 and more accurately in the similarity matrix analysis (Table 2 and 3). The maximum nucleotide difference for the 16s rRNA gene ranges from 0.1% to 1.4% whilst for the *gyrB *gene it ranges from 3.5% to 16.1%. The differences in nucleotide divergence between the two genes is due to *gyrB *having a higher rate of base substitution (0.7–0.8% per 1 million years) when compared to 16s rRNA (1% per 50 million years) [15].

**Figure 4:**
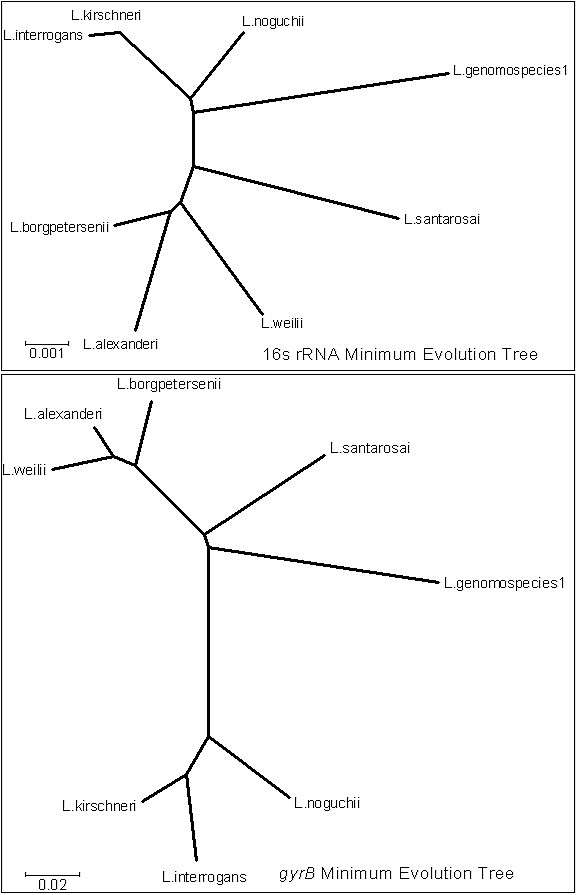
Minimum evolution trees of the *gyrB *and 16s rRNA gene created using the MEGA V3.1 Software package.

In addition to the cluster analysis, minimum evolution trees for 16s rRNA and *gyrB *were constructed using 1000 bootstrap replications (Figure 4). The trees have nearly identical topology except the evolution of *L. kirschneri *and *L*. *interrogans *in the *gyrB *gene is quite distinctly different to that of the evolution pattern in the 16s rRNA gene. The changes in topology signifies that the *gyrB *gene in pathogenic *Leptospira *has undergone significant evolutionary divergence compared to that of the 16s rRNA gene and this result is consisted with research conducted in other bacterial genera including the preliminary work conducted by Kawabata *et al. *with *Leptospira *[7-9,11,12,14-18].

### Identification of *Leptospira *clinical isolates using *gyrB *PCR

To validate the use of *gyrB *as an alternative target to 16s rRNA, a comparison study was performed using 50 unidentified clinical isolates all from human sources. The *gyrB *sequences of the unknown isolates were compared to those deposited on GenBank using a BLASTn search. Confirmation of the *gyrB *result was performed using 16s rRNA sequencing and BLAST analysis as described in the methods. There was 100% agreement between the 16s rRNA and *gyrB *gene sequencing for the identification of pathogenic *Leptospira *species. Within the 50 isolates tested there was found to be the following number of species; *L. alexanderi *(1 isolate), *L. weilli *(10 isolates), *L. borgpetersenii *(10 isolates) and *L. interrogans *(29 isolates). The advantage of using the *gyrB *gene over the 16s rRNA gene is that during the BLASTn searches on GenBank, the score values were generally higher and the E values generally lower for the predicted species when compared to the 16s rRNA gene BLASTn searches allowing for greater confidence in the final result.

## Conclusion

We have developed and validated a conventional and real-time PCR method for the amplification of *gyrB *gene from pathogenic *Leptospira*. When compared to the 16s rRNA gene, the *gyrB *gene shows greater evolutionary divergence and an alternate evolutionary topology for *L. kirschneri *and *L. interrogans *using minimum evolution analysis. Additionally the greater divergence of the *gyrB *gene makes it more amendable to the identification of pathogenic *Leptospira *either through sequencing as shown in this study or in the future by Real-time PCR using DNA probe technology.

## Methods

### *Leptospira *strains and DNA extraction

In total, 37 reference strains from the eight pathogenic *Leptospira *species and 50 clinical *Leptospira *isolates from human sources were obtained from the WHO/FAO/OIE Collaborating Centre for Reference & Research on Leptospirosis, Brisbane, Australia. Genomic DNA was extracted by the following method: 500 μL of Ellinghausen McCullough Johnson Harris (EMJH) media containing actively growing *Leptospira *was centrifuged in a micro-centrifuge tube at 12,000 g for 5 min. The supernatant was removed and the pellet re-suspended in 400 μL of 1× TE Buffer (10 mM Tris, 1 mM EDTA, pH 8.0). This suspension was boiled for 10 min and then centrifuged at 12,000 g for 5 min.

### *gyrB *amplification: Conventional PCR

PCR primers were developed from the two available Leptospira interrogans genome sequences: NC_005823[19,20] and NC_004342[21] using Primer Premier 5.0 (Premier Biosoft) to amplify a 502 base pair (bp) fragment of the *gyrB *gene. PCR amplification was performed in a final volume of 25 μL using 1 × PCR buffer, 2.5 mM Magnesium Chloride (MgCl_2_), 200 μM dNTPs, 12.5 pmol of oligonucleotides; 2For and 504Rev (Table 4), one unit of AmpliTaq Gold, 2 μl of DNA extract and double distilled water (ddH_2_O) to make up the final volume. Thermal cycling was as following: Initial denaturation at 94°C for 10 min, followed by 35 cycles of 94°C for 30 s, 60°C for 30 s and 72°C for 30 s with a final extension at 72°C for 10 min. 5 μL of PCR product was electrophoresis on 1.5% agarose gel at 80 V for 60 min (Figure 1).

### *gyrB *amplification: Real-time PCR

Real-time amplification of the *gyrB *gene was performed in a total volume of 20 μL containing 2 μL of Fast-Start Sybr green mix (Roche), 2.4 μL of 25 mM MgCl_2_, 10 pmol of oligonucleotides; 2For and 504Rev, 2 μL of template DNA and ddH_2_O to make up the final volume. Thermal cycling was performed on a LightCycler real-time thermalcycler (Roche) using the following program: initial denaturation at 95°C for 10 min and 40 cycles of 95°C for 10 s, 60°C for 20 s and 72°C for 20 s. Fluorescence readings were taken at the end of each extension cycle in the F1 (FAM/Sybr green) channel. Melting curve analysis was performed by heating the PCR product from 60°C to 95°C and monitoring the fluorescence change every 0.2°C. The melting temperature or Tm was calculated by calculated on the initial fluorescence curve (F1/I) by plotting the negative derivative of fluorescence over temperature versus temperature (-dF1/dT versus T). Amplified products were removed before sequencing from the capillaries by uncapping and inverting the capillary in a micro-centrifuge tube and centrifuging at 1,500 g for 10 s.

### 16s rRNA amplification

16s rRNA amplification was performed in a final volume of 25 μL containing 1× PCR buffer, 2.0 mM of MgCl_2_, 200 μM dNTPs, 10.0 pmol of oligonucleotides; FD1MOD and 13R (Table 4), one unit of AmpliTaq Gold, 2 μl of DNA extract and (ddH_2_O) to make up the final volume. Thermal cycling was as following: Initial denaturation at 94°C for 10 min, followed by 35 cycles of 94°C for 30 s, 55°C for 30 s and 72°C for 30 s with a final extension at 72°C for 10 min. Agarose electrophoresis was performed as above. The 1382 bp product were sequenced as described below in both the forward and reverse directions using the original primers and with internal primers; 515F, 91e, 11e, 16s1RRB, 907R and 342R (Table 4).

### DNA Sequencing

Excess primers and dNTP's were removed from the remaining PCR product using the following enzymatic method: 2.5 μl of 10× Antarctic phosphatase buffer (New England Biolabs, NEB), 10 units of Exonuclease I, *E. coli *(Fermentas), 2.5 units of Antarctic phosphatase (NEB) and 1.5 μl of ddH_2_O were added to each sample. The PCR product plus enzyme mix were incubated at 37°C for 45 min followed by 85°C for 15 min to inactivate the enzyme. DNA sequencing was performed using the Big Dye Terminator (BDT) sequencing version 3.1 (Applied Biosystems) with the following modifications: each 20 μL reaction contained 0.5 μL of BDT mix (1/16^th ^dilution in final volume), 3.75 μL of 5× dilution buffer, 3.2 pmol of primer, 5–10 ng of DNA and ddH_2_O to make up the final volume. Cycle sequencing was performed using 33 cycles of 95°C for 10 s, 50°C for 10 s and 60°C for 4 min. The cycle sequencing products were purified using the sodium acetate/alcohol precipitation method as per manufacturers' instructions (Applied Biosystems). The purified products were forwarded to the Griffith university DNA sequencing facility (GUDSF), Brisbane, Australia for capillary electrophoresis using the ABI 3130 × l instrument. The sequences were assembled and trimmed to a minimum of two contiguous sequences using the Vector NTI software (Invitrogen).

### Data Analysis

Analysis of the DNA sequences was performed using the Bionumerics (Applied maths) and MEGA version 3.1 software packages [22]. 37 reference sequences for both gyrB and 16s rRNA were deposited on GenBank (Table 1). Unknown isolates were submitted to a nucleotide Basic Local Alignment Search Tool (BLASTn) [23] search available at the National Centre for Biotechnology Information (NCBI) website [24].

## Competing interests

The author(s) declare that they have no competing interests.

## Authors' contributions

AS was responsible for design of the study, conducting the molecular experiments and the preparation of the manuscript. MD and MS provided laboratory support by providing culture, maintaining culture collections and contributed to the editing of the manuscript. LS approved the research study/funding, provided intellectual input and contributed to the editing of the manuscript. All authors have read and approved the final manuscript.

## Figures and Tables

**Table 1:** Details of *gyrB *and 16s rRNA sequences from the pathogenic *Leptospira *species deposited on GenBank from this study.

Species	Serovar	Strain	GenBank Accession number – gyrB sequence	GenBank Accession number – 16s rRNA sequence
*L. interrogans*	Australis	Ballico	AY896758	DQ991464
	Djasiman	Djasiman	AY896757	DQ991465
	Szwajizak	Szwajizak	AY896756	DQ991466
	Kremastos	Kremastos	AY896755	DQ991467
	Hardjo	Hardjoprajitno	AY896754	DQ991468
	Bataviae	Swart	AY896753	DQ991469
	Copenhageni	M20	AY896747	DQ991470
	Medanesis	Hond HC	AY896746	DQ991471
	Canicola	Hond Utrecht IV	AY896745	DQ991472
	Zanoni	Zanoni	AY896744	DQ991473
	Pomona	Pomona	AY896738	DQ991474
*L. kirschneri*	Cynopteri	3522C	AY896759	DQ991475
	Agogo	Agogo	DQ641396	DQ991476
	Bafani	Bafani	DQ641397	DQ991477
	Butembo	Butembo	DQ641398	DQ991478
	Ratnapura	Wumalasena	DQ641399	DQ991479
*L. *genomospecies 1	Pingchang	80–412	AY896752	DQ991480
*L. alexanderi*	Mengla	A85	AY896751	DQ991481
	Manzhuang	A23	AY896750	DQ991482
*L. borgpetersenii*	Javanica	Veldrat Batavia 46	AY896743	DQ991483
	Ballum	Mus 127	AY896742	DQ991484
	Tarassovi	Perepelitsin	AY896738	DQ991485
*L. weilii*	Celledoni	Celledoni	AY896740	DQ991486
	Hekou	H27	DQ641409	DQ991487
	Langati	M39090	DQ641410	DQ991488
	Sarmin	Sarmin	DQ641402	DQ991489
	Vughia	LT89-68	DQ641411	DQ991490
*L. santarosai*	Alexi	HS-616	AY896749	DQ991491
	Shermani	1342K	AY896739	DQ991492
	Alice	Alice	DQ641405	DQ991493
	Bakeri	LT 79	DQ641406	DQ991494
	Kobbe	CZ 320	DQ641407	DQ991495
	Weaveri	CZ 390	DQ641408	DQ991496
*L. noguchii*	Cristobali	1996K	DQ641401	DQ991497
	Claytoni	1348U	DQ641400	DQ991498
	Huallaga	M7	DQ641403	DQ991499
	Panama	CZ 214	DQ641404	DQ991500

**Table 2:** Similarity matrix constructed using the 16s rRNA DNA sequences from pathogenic Leptospira *species*.

*Leptospira *species Details	Similarity Matrix (%)							
*L. weilii *sv. Celledoni strain Celledoni	100							
*L. alexanderi *sv. Manzhaung strain A23	99.0	100						
*L. borgpetersenii *sv. Ballum strain Mus 127	99.6	99.3	100					
*L. santarosai *sv. Shermani strain 1342K	99.1	98.6	99.2	100				
*L. *genomospecies 1 sv. Pingchang strain 80–412	98.9	98.7	99.0	98.6	100			
*L. interrogans *sv. Australis strain Ballico	99.1	98.9	99.3	98.8	99.1	100		
*L. kirschneri *sv. Cynopteri strain 3522C	99.2	99.0	99.4	98.9	99.2	99.9	100	
*L. noguchii *sv. Panama strain CZ 214K	99.2	98.8	99.3	99.1	99.1	99.4	99.5	100

**Table 3:** Similarity matrix constructed using the gyrB DNA sequences from pathogenic Leptospira *species*.

*Leptospira *species Details	Similarity Matrix (%)							
*L. weilii *sv. Celledoni strain Celledoni	100							
*L. alexanderi *sv. Manzhaung strain A23	96.5	100						
*L. borgpetersenii *sv. Ballum strain Mus 127	94.3	96.1	100					
*L. santarosai *sv. Shermani strain 1342K	88.6	90.6	90.6	100				
*L. *genomospecies 1 sv. Pingchang strain 80–412	86.8	87.0	88.2	87.4	100			
*L. interrogans *sv. Australis strain Ballico	84.7	85.1	84.5	85.5	83.9	100		
*L. kirschneri *sv. Cynopteri strain 3522C	85.9	86.1	85.3	86.8	84.3	95.2	100	
*L. noguchii *sv. Panama strain CZ 214K	85.7	85.7	85.3	86.4	84.3	92.5	92.9	100

**Table 4:** Oligonucleotides used in this study.

Assay	Use	Oligonucleotide	Sequence (5'–3')	Reference
*gyrB *– Conventional and real-time PCR.	Amplification and sequencing	2For	TGAGCCAAGAAGAAACAAGCTACA	This study
		504Rev	MATGGTTCCRCTTTCCGAAGA	
16s rRNA gene	Amplification and sequencing	FD1MOD	AGAGTTTGATCYTGGYTYAG	[25]
		13R	AGGCCCGGGAACGTATTCAC	[26]
	Sequencing	515F	GTGCCAGCAGCCGCGGTAA	[26]
		91e	TCAAAGGAATTGACGGGGGC	[26, 27]
		11e	GAGGAAGGTGGGGATGACG	[27]
		16s1RRB	CTTTACGCCCARTRAWTCCG	[28]
		907R	CCGTCAATTCCTTTRAGTTT	[29]
		342R	CTGCTGCSYCCCGTAG	[29]
